# Supplementary material for: Visceral Obesity in Non-Small Cell Lung Cancer
Source: Cancers (Basel). 2022 Jul 15;14(14):3450. doi: 10.3390/cancers14143450 (PMC9315749; doi:10.3390/cancers14143450)
Supplement: Supplementary file 1 [file cancers-14-03450-s001.zip › cancers-1800727-supplementary.pdf]

Supplemental material

Nitsche L, Vedire Y, Kannisto E, et al. Visceral obesity in non-small cell lung cancer. 2022.

Content

1. Table S1. Characteristics of the examined population.
2. Table S2. STROBE statement with items recommended for reports of cohort studies.
3. Table S3. Sex-specific characteristics of the population examined at the L3 vertebral level.

Table S1. Characteristics of the examined population[a]

Age at tumor diagnosis (years)	*Sex*	*Race*	*Tobacco smoking history at tumor diagnosis*	*Year of surgery/biopsy of tumor*	*Pathological tumor stage*	*Tumor histology*	*Body mass index*	*Year of CT scan*	*Vertebral level for fat area measurement*	*Subcutaneous fat area (mm^2)*	*Visceral fat area (mm^2)*
72	F	White		2018	4	Other	20.4	2018	L3	6499	3089
78	F	White	Former	2013	2	SCC	14.6	2013	L3	2177	4121
75	F	White	Current	2013	1	AC	24.0	2013	L3	17475	15770
53	F	White	Former	2015	1	AC	30.4	2015	L3	32227	16338
86	F	White	Never	2014	1	AC	26.6	2013	L3	17862	18054
64	F	White	Current	2010	1	AC	30.1	2010	L3	27728	21154
81	F	White	Former	2014	2	SCC	22.7	2014	L3	11353	18428
74	F	White	Former	2009	1	SCC	37.6	2009	L3	29109	30475
76	F	White		2016	1	AC	20.5	2016	L3	15404	7277
64	F	White	Former	2012	1	Other	36.2	2012	L3	44510	17011
82	M	White	Former	2011	2	SCC	22.3	2011	L3	9223	18253
82	F	White	Former	2014	2	AC	28.8	2014	L3	29122	15059
84	F	White	Current	2015	2	AC	36.1	2015	L3	37962	23888
76	M	White	Former	2013	4	Other	32.1	2013	L3	20048	36466
65	F	White		2017		SCC	26.6	2017	L3	19009	11031
75	F	White	Never	2009	1	SCC	30.3	2009	L3	29145	19882
55	F	White	Former	2019	1	Other	39	2019	L3	46300	35000
77	F	White	Former	2013	2	AC	30.8	2013	L3	32698	17525
69	M	White	Current	2012	1	AC	26.0	2012	L3	11262	21129
76	F	Black	Never	2009	2	SCC	25.9	2009	L3	19086	13221
90	M	White	Former	2013	2	AC	22.6	2013	L3	15096	17634
67	M	White	Former	2010	3	SCC	37.7	2010	L3	36345	48093
84	M	White	Former	2009	2	SCC	26.9	2009	L3	19996	16169
73	F	White	Former	2012	3	SCC	39.9	2012	L3	38409	37480
75	M	White		2017	4	AC	24.1	2018	L3	7856	31540
81	F	White	Never	2013	1	AC	34.5	2013	L3	41809	28037
72	F	White	Current	2010	1	AC	25.4	2010	L3	16159	17343
61	F	White	Former	2013	1	AC	21.1	2013	L3	8524	3318
71	M	White	Former	2016	2	SCC	32.5	2016	L3	36400	37800
61	F	White	Former	2013	3	SCC	26.8	2013	L3	26804	16024
72	F	White	Never	2010	1	Other	28.9	2010	L3	29361	17657
75	F	White	Former	2010	3	AC	26.6	2010	L3	28985	14764
76	F	White	Former	2010	1	AC	29.6	2010	L3	29278	18161
63	F	White	Current	2015	2	SCC	19.2	2015	L3	3413	2090
64	F	White	Former	2013		SCC	34.2	2013	L3	12469	6687
55	F	White	Former	2011	2	AC	30.4	2011	L3	34728	9385
73	M	White	Current	2010	1	AC	21.0	2010	L3	7215	5108
71	M	Black	Current	2015	1	AC	27.1	2015	L3	15753	17588
70	F	White	Former	2015	2	SCC	20.3	2015	L3	16188	7699

68	F	White	Never	2008	2	AC	34.5	2008	L3	29732	13808
73	F	White	Former	2014	1	AC	37.1	2014	L3	30080	25125
73	F	White	Never	2014	2	AC	27.7	2013	L2	20101	27357
71	M	White	Former	2010	1	AC	29.2	2009	L3	22532	23640
69	M	White	Former	2009	1	AC	27.9	2009	L1	12898	14739
79	F	White	Former	2010	1	AC	25.1	2010	L3	18349	10667
55	F	Black	Former	2013	1	AC	28.3	2013	L3	15736	11751
80	M	White	Former	2015	3	AC	27.3	2015	L3	19784	31324
49	F	White	Never	2009	2	Other	16.7	2009	L3	1846	1567
51	M	Black	Current	2013	1	SCC	21.7	2013	L3	12525	5567
76	M	White	Former	2012	1	Other	24.5	2011	L3	17295	22263
58	M	Other		2017	1	AC	25	2017	L3	11255	9129
73	M	Black	Former	2011	1	SCC	24.5	2011	L3	10887	9047
56	F	White	Former	2013	4	AC	27.1	2013	L3	27357	11890
57	F	White	Current	2012	1	AC	25.3	2012	L3	25666	12632
57	M	Black	Former	2013	2	AC	26.5	2013	L3	16781	28260
66	F	White	Current	2009	1	AC	28.0	2009	L3	29644	14104
66	M	White		2018		AC	27.3	2018	L3	17534	25378
56	F	White	Former	2013	1	SCC	24.2	2013	L3	22594	14322
81	F	White	Current	2015	1	AC	24.1	2015	L3	5330	5278
71	F	White	Former	2014	1	AC	24.3	2014	L3	18001	12104
72	F	White		2017	4	SCC	35.4	2017	L3	38699	22255
60	M	White	Current	2009	1	AC	27.6	2009	L3	16287	17004
80	M	White		2017		AC	29.2	2017	L3	27257	31210
61	M	White		2018		SCC	36	2018	L3	30670	41683
61	F	White	Former	2014	1	AC	30.1	2014	L3	19296	21351
58	F	White	Current	2010	2	AC	19.7	2010	L3	10993	6212
78	F	White	Current	2014	1	SCC	18.9	2014	L3	6770	8166
63	F	White	Former	2012	1	AC	22.5	2012	L3	14350	6952
82	F	White	Never	2011	1	AC	19.6	2011	L3	13306	4267
75	M	White	Never	2013	1	AC	31.8	2013	L3	24548	33558
69	F	White	Former	2017	2	Other	36	2017	L3	50900	21700
79	F	Black	Current	2011	1	SCC	17.8	2011	L3	4577	3736
78	F	White		2018	4	Other	34.5	2018	L3	37145	19819
62	M	White	Former	2014	2	SCC	39.9	2014	L3	40273	44485
76	F	White	Never	2014	1	AC	19.3	2014	L3	2725	3725
65	F	White	Former	2010	1	Other	25.4	2010	L3	20029	23838
65	M	White	Former	2012	2	AC	35.5	2012	L3	26336	57574
80	M	Other	Former	2013	2	AC	35.6	2012	L3	25386	58546
70	F	Black		2018	4	AC	35.4	2018	L3	45180	13423
87	F	White	Former	2015	1	AC	27.3	2015	L3	23423	20487
74	M		Former	2015	1	AC	28.1	2015	L3	20134	34692
65	F	Black	Former	2017	2	AC	37.8	2016	L3	39200	31600
74	M	White	Never	2011	1	AC	28.7	2011	L3	19822	26936
76	M	White	Former	2014	1	AC	40.9	2014	L3	16094	33466
40	F	White	Never	2015	1	AC	41.9	2015	L3	45709	14900
69	F	White	Current	2012	2	SCC	23.6	2012	L3	20462	17623
81	M	White		2018		AC	24	2017	L3	16582	28647
74	F	Black	Current	2015	2	SCC	23.9	2015	L3	22522	9565
60	F	Black	Current	2009	1	AC	25.5	2009	L3	27354	9021
65	F	White	Former	2012	1	AC	35.8	2012	L3	39962	20996

78	M	White	Former	2013	1	SCC	30.8	2013	L3	37609	28228
74	F	White	Former	2013	1	SCC	32.1	2013	L3	25981	21456
85	F	White	Former	2015	2	Other	23.1	2015	L3	13288	4481
72	F	White	Former	2012	2	AC	39.0	2012	L3	62744	30103
65	M	White	Former	2013	1	AC	31.8	2012	L3	15486	54124
76	F	White	Former	2010	1	SCC	28.5	2010	L3	31459	16027
71	F	White	Former	2009		Other	33.6	2009	L3	32913	21914
81	M	Black		2018		SCC	29	2018	L3	19765	27743
83	F	White		2016		AC	26.8	2016	L3	22411	30841
73	F	White		2018	3	AC	24.5	2018	L2	13934	11791
63	M	White	Current	2014	1	SCC	20.8	2014	L3	10455	23209
73	F	White	Current	2015	1	AC	24.6	2015	L3	18149	16920
80	F	White	Former	2011	1	AC	20.5	2011	L3	8966	3335
71	F	White	Current	2015	1	SCC	36.8	2015	L3	43331	17812
76	F	White	Former	2013	1	SCC	22.3	2012	L3	15323	5476
66	F	White		2017		AC	28.5	2017	L3	25995	13235
77	F	White	Former	2013	1	AC	29.1	2013	L3	12755	17897
69	F	Black		2017		AC	19.2	2017	L3	9302	5573
71	M	White	Former	2013	1	SCC	36.8	2013	L3	29377	31970
60	F	Black	Former	2015	1	SCC	43.6	2015	L3	46260	19594
58	F	White	Former	2009	1	AC	32.1	2009	L3	33201	23448
71	M	White		2017	4	AC	22.3	2017	L3	16007	10245
66	F	White	Current	2014	1	AC	22.3	2014	L3	13652	8285
79	M	Black		2018		Other	20	2018	L3	7541	7753
61	M	White		2014	2	Other	33.1	2014	L3	27727	28470
55	F	White	Current	2014	1	AC	18.0	2014	L3	11104	7919
63	M	White	Former	2011	1	AC	24.7	2011	L3	23349	17918
73	M	White	Current	2013	2	SCC	31.6	2013	L3	27905	32118
56	F	White	Current	2010	1	AC	22.4	2010	L3	17609	8185
57	M	White		2017		Other	29	2017	L3	13813	39450
68	M	White	Current	2013	3	SCC	31.0	2013	L3	24319	32906
85	M	Black	Former	2015	1	AC	26.9	2015	L3	15395	18868
63	F	White	Current	2012	2	AC	36.9	2012	L3	38840	24192
76	F	White	Never	2010	1	AC	23.2	2010	L3	6826	8208
66	F	White	Never	2013	2	AC	27.1	2013	L3	18042	5142
46	F	Black	Current	2013	2	AC	20.0	2013	L3	11532	4136
70	M	White	Former	2010	2	Other	31.4	2010	L3	22885	28792
86	F	White	Former	2009		AC	23.4	2009	L3	13465	8315
53	F	Black	Current	2010	1	AC	36.9	2009	L3	47523	16285
60	M	Black	Former	2011	1	AC	31.0	2011	L3	29135	10110
76	M	White	Former	2014	1	AC	26.4	2014	L3	14156	34732
56	M	White	Former	2012	2	AC	25.0	2012	L3	16936	13542
70	F	White		2018	1	AC	26.5	2018	L3	29178	26182
74	M	White	Former	2012	1	AC	25.1	2012	L3	15683	11016
64	F	White	Former	2014	1	SCC	25.3	2014	L3	14816	4408
77	M	White		2018	4	SCC	27.2	2018	L3	21284	40666
74	M	White	Former	2011	2	Other	28.8	2011	L3	10966	58095
74	M	White	Former	2010	2	SCC	23.4	2010	L3	11695	11788
72	F	White	Former	2011	1	AC	26.9	2011	L3	21697	15912
61	M	White	Current	2008	1	AC	35.3	2008	L3	28731	41376
88	M	White		2018	1	SCC	22.6	2018	L2	10549	15489

68	M	White	Never	2011	2	AC	28.9	2010	L3	17922	24514
79	M	White	Former	2010	1	SCC	26.3	2010	L3	14682	35019
70	M	White	Former	2010	1	SCC	35.1	2010	L3	27178	47449
61	M	White	Former	2013	3	SCC	21.7	2013	L3	4783	8838
74	M	White	Former	2009	1	SCC	35.4	2009	L3	24226	22436
63	F	White	Former	2012	1	AC	25.6	2012	L3	23477	19015
63	M	White	Former	2011	1	SCC	26.6	2010	L3	9673	21845
53	M	White	Former	2011	1	AC	19.5	2011	L3	14879	24519
56	M	White	Former	2013	1	SCC	28.0	2013	L3	26485	14903
64	F	White	Current	2019	1	AC	22.2	2019	L3	12600	17000
59	F	White	Former	2011	1	AC	29.8	2011	L3	21100	5799
66	M	White		2018	1	SCC	24.5	2018	L3	14493	21392
74	M	White	Current	2015	1	AC	21.9	2015	L3	13856	17619
46	M	White	Current	2008		AC	22.7	2008	L3	7186	11461
74	F	White	Never	2008		AC	28.0	2008	L1	13896	18201
56	F	White	Never	2009	1	AC	20.4	2009	L3	19116	6386
85	F	White		2008	1	AC	23.2	2008	L3	13302	6939
83	F	White	Never	2008	1	AC	25.0	2008	L3	15250	7560
81	F	White	Current	2009		SCC	27.1	2009	L3	18416	13686
32	F	White	Former	2009		Other	32.8	2008	L3	33169	10441
51	F	White	Former	2008	2	AC	22.0	2008	L3	16301	9655
64	F	White	Former	2008	1	AC	30.5	2008	L3	45510	21447
33	F	White	Never	2008		Other	21.0	2008	L3	15469	5939
55	F	White	Current	2008		AC	26.7	2008	L3	24745	12743
71	M	Black	Former	2009	3	AC	20.1	2009	L3	10322	8466
66	M	White	Former	2010	1	AC	21.2	2009	L3	16593	16163
81	F	White	Current	2009	1	SCC	17.5	2008	L3	3337	4912
75	M	White	Former	2013	1	AC	27.0	2013	L3	17314	25013
45	F	White	Former	2009		Other	36.0	2009	L3	41999	19749
71	M	White	Current	2019		SCC	30.3	2019	L3	24300	34500
60	F	White	Former	2008	1	AC	41.1	2008	L3	54848	44320
79	F	White	Never	2008	1	AC	29.8	2008	L3	20611	20505
74	M	White	Never	2009	1	AC	22.4	2009	L3	6876	6771
84	M	White	Former	2009	1	SCC	28.6	2008	L3	15037	36727
66	F	White	Former	2009	1	AC	38.6	2009	L3	48810	29023
64	F	White	Former	2009	4	AC	38.8	2008	L3	34804	31498
49	F	White	Former	2009	1	AC	35.5	2008	L3	47419	19285
52	F	White	Former	2009	4	AC	19.8	2008	L3	7997	7758
75	F	White	Former	2009	1	AC	24.4	2009	L3	13359	13453
74	M	White	Former	2009	1	SCC	32.3	2008	L1	9892	38217
53	M	Black	Former	2009	1	SCC	30.8	2009	L3	35040	24292
64	M	White	Former	2009	1	AC	35.2	2009	L3	29682	32926
68	M	White	Former	2009	1	AC	30.5	2009	L3	28971	21085
69	F	White	Former	2009	1	SCC	26.7	2009	L3	23413	11447
68	F	White	Former	2009	1	AC	34.6	2009	L1	21811	18572
81	F	White	Former	2009	1	SCC	33.3	2008	L3	34729	24097
82	F	Black	Former	2009	1	SCC	27.4	2009	L3	27989	7537
66	M	White	Former	2009	1	AC	28.4	2009	L3	20068	19701
68	M	White	Never	2012	2	AC	28.0	2012	L3	17864	14381
59	M	White	Former	2009	2	SCC	20.7	2009	L1	6047	8076
62	F	White	Former	2012	1	AC	25.4	2012	L3	20754	7334

68	F	White	Former	2009	1	AC	25.6	2009	L3	15437	15505
70	M	White	Former	2009	3	SCC	28.2	2009	L3	9416	42736
52	F	White	Current	2009	1	SCC	23.1	2009	L3	16355	7758
66	M	White	Former	2009	3	AC	38.1	2009	L3	36043	46951
56	F	White	Former	2015	1	AC	21.1	2014	L3	6521	4420
74	M	White	Former	2009	1	AC	26.2	2009	L3	14366	32938
66	F	Other	Former	2009	2	AC	34.6	2009	L3	39393	27935
54	M	White	Current	2009	3	SCC	22.3	2009	L3	5217	4763
80	M	White	Former	2009	2	Other	28.3	2009	L3	29136	27311
62	F	White	Current	2009	1	AC	26.1	2009	L3	29529	9035
84	M	White	Former	2009	2	SCC	25.8	2009	L3	11828	23581
62	F	White	Former	2009	1	AC	19.1	2009	L3	8638	4449
74	F	White	Former	2009	1	AC	19.7	2009	L3	5010	3622
82	F	White	Former	2009	1	AC	24.7	2009	L3	16095	10035
81	F	White	Former	2009	1	SCC	25.2	2009	L3	17575	11121
77	F	White	Current	2009	1	SCC	23.8	2009	L3	18567	5401
58	F	White	Former	2009	1	Other	22.4	2009	L3	10811	8553
86	F	White	Former	2009	3	SCC	25.7	2009	L3	12998	14375
51	M	White	Current	2009	2	AC	21.5	2009	L3	9435	7376
69	M	White	Former	2015	2	AC	32.2	2015	L3	30010	30226
82	F	White	Current	2009	1	SCC	16.7	2009	L3	2962	4889
52	F	White	Current	2014	1	AC	23.5	2014	L3	26440	18812
66	F	White	Former	2009	1	AC	23.1	2009	L3	15815	9001
76	F	White	Former	2011	3	SCC	25.8	2011	L2	19506	5008
68	M	Asian	Never	2014	2	AC	27.1	2014	L3	19030	29406
25	F	White		2009	1	Other	20.9	2009	L3	6162	2381
78	F	White	Former	2012	1	SCC	28.1	2012	L3	16202	13813
74	F	White	Former	2010	3	AC	32.3	2010	L2	20739	34422
59	F	White	Current	2010	1	AC	20.3	2009	L3	6681	2350
73	M	White	Former	2010	1	SCC	17.6	2009	L3	3831	3258
73	F	White	Former	2015	1	SCC	27.0	2015	L3	16562	12533
79	F	White	Never	2009		Other	25.0	2009	L3	21951	19993
77	M	White	Former	2010	1	AC	25.1	2009	L3	13237	19483
79	M	White	Current	2010	1	SCC	25.5	2010	L3	16337	24121
70	F	White	Former	2010	1	AC	53.0		L3	16697	7548
49	M	White	Current	2010	2	SCC	32.8	2009	L3	15702	24728
74	M	White	Former	2010		Other	31.6	2010	L3	25598	34110
75	M	White	Current	2013	1	AC	29.4	2013	L3	14967	27415
79	M	White	Former	2010	2	AC	39.5	2010	L3	26068	42003
55	M	White	Current	2010	1	SCC	27.8	2010	L3	11649	31176
74	M	White	Former	2010	1	AC	22.9	2010	L3	11655	5637
73	F	White	Former	2010	2	AC	25.2	2010	L3	12646	16429
80	M	White	Former	2010	2	SCC	25.9	2010	L2	9985	23148
71	F	White	Current	2012	1	SCC	22.0	2012	L3	9100	12677
76	F	White	Never	2010	2	AC	21.4	2010	L3	12173	10197
74	F	White	Former	2011	1	SCC	32.3	2011	L3	38259	19004
62	F	White	Never	2010	1	AC	56.2	2009	L3	65366	36816
62	M	White	Former	2010	1	AC	24.1	2010	L3	14681	21188
59	M	White	Current	2010	3	Other	21.0	2010	L2	7139	5120
81	F	White	Former	2011	1	AC	25.1	2011	L3	13888	14891
62	F	White	Former	2010	1	SCC	21.3	2010	L3	20074	19919

63	F	White	Former	2010	1	AC	29.6	2010	L3	22979	17530
60	F	White	Current	2010	1	SCC	36.1	2010	L3	40659	20975
70	F	White	Current	2010	1	SCC	24.4	2010	L3	9055	7846
60	M	White	Former	2010	2	SCC	21.9	2010	L3	4949	5443
58	F	White	Current	2010	2	AC	27.9	2010	L3	36374	14081
70	M	White	Current	2010	1	SCC	23.3	2010	L3	10578	14875
78	F	White	Current	2010	1	AC	29.2	2010	L3	27432	16509
69	M	White		2017	1	AC	32.7	2017	L3	36693	17351
63	M	White	Former	2014	1	AC	29.1	2014	L3	21200	34115
54	M	White	Current	2010	3	SCC	21.7	2010	L2	9791	16444
73	F	White	Former	2010	1	SCC	28.8	2010	L3	22947	27239
45	F	White	Current	2010	1	AC	19.6	2010	L3	4619	5216
57	M	White	Current	2015	1	AC	29.8	2015	L3	18835	31683
51	M	White	Current	2010	2	Other	33.9	2010	L3	32152	24911
40	F	White	Former	2010	3	AC	31.7	2010	L3	25577	7793
59	M	White	Current	2015	1	AC	24.2	2015	L3	18638	11321
58	M	Black	Current	2012	1	AC	28.2	2012	L3	21346	17398
74	F	Black	Former	2011	1	AC	26.3	2011	L2	13558	13378
78	F	White	Former	2011	1	AC	32.4	2011	L3	24626	21514
65	F	White	Former	2011	1	AC	22.4	2011	L3	6731	5522
59	M	White	Former	2011	1	AC	24.5	2011	L3	11732	24096
81	M	White	Former	2012	1	Other	28.8	2011	L3	21909	33497
69	F	White	Former	2011	2	AC	34.0	2011	L3	27616	28045
75	M	White		2018	4	AC	31.2	2018	L3	17651	30423
53	M	White	Former	2014	2	AC	21.0	2014	L3	10639	13541
53	F	Black	Current	2012	1	AC	24.0	2012	L3	14779	4286
43	F	White	Current	2010	1	AC	17.9	2010	L3	10950	3711
72	F	White	Former	2010	1	AC	26.2	2010	L3	21154	8249
67	M	White	Current	2010	1	AC	23.3	2010	L3	17058	11388
55	F	White	Former	2011	1	AC	35.8	2011	L3	40216	12173
61	F	White	Former	2010	2	AC	31.1	2010	L3	36329	13778
78	F	White	Former	2010	2	SCC	23.7	2010	L3	17270	13949
71	M	White	Current	2010	1	SCC	27.1	2010	L3	23137	19539
57	M	White	Former	2010	1	AC	23.9	2010	L3	6358	4980
49	F	White	Current	2010	2	AC	28.6	2010	L3	34542	9778
50	F	White	Current	2014	3	SCC	19.5	2014	L3	13819	4071
81	F	White	Never	2010	1	AC	43.4	2010	L3	43666	16962
70	F	White	Current	2010	2	AC	25.7	2010	L3	19579	7877
67	M	White	Former	2010	1	SCC	28.5	2010	L3	18929	33309
61	F	White	Current	2010	1	SCC	29.7	2010	L3	24529	5640
72	F	Black	Current	2010	1	AC	17.9	2010	L3	6642	2685
71	F	White	Former	2010	1	SCC	26.7	2010	L3	24891	29540
74	M	White	Former	2010	1	SCC	28.2	2010	L3	17820	18341
62	M	White	Current	2010	3	SCC	30.6	2010	L3	20496	21723
68	F	White		2018	4	AC	28.2	2018	L3	23692	16770
60	M	White	Never	2010	2	AC	29.5	2010	L3	19213	40439
63	F	White	Former	2010	3	Other	40.4	2010	L3	39201	32576
53	F	Black	Current	2010	1	SCC	22.8	2010	L3	13273	4967
81	M	White	Former	2010	1	SCC	26.2	2010	L3	11880	26454
70	M	White	Former	2011	1	Other	25.7	2011	L3	11382	24535
57	F	Other	Never	2010	2	Other	18.7	2011	L3	9312	2342

79	F	Black	Former	2013	1	SCC	27.1	2013	L3	20288	7625
58	F	White	Former	2010	1	AC	27.4	2010	L3	27923	13998
48	M	White	Current	2010	2	AC	22.3	2010	L3	12083	8750
71	M	White	Never	2010	1	AC	26.1	2010	L3	14874	18367
73	F	White	Former	2011	3	AC	30.5	2011	L3	38013	31169
76	F	Black		2017		AC	24.8	2017	L3	17671	4644
66	M	White	Former	2013	1	AC	30.7	2013	L3	27640	16298
51	F	White	Current	2010	1	AC	37.7	2010	L3	41331	35872
53	F	Black	Never	2011	1	AC	25.2	2011	L3	24575	7382
87	M	White	Former	2011	1	AC	24.6	2010	L3	15821	27228
43	M	White	Current	2011	2	SCC	27.5	2010	L3	15750	18524
71	M	White	Former	2011	1	AC	25.6	2010	L3	14996	22986
67	M	White	Former	2010	1	AC	19.6	2010	L3	8660	2745
72	F	White	Former	2010	2	SCC	38.5	2010	L3	59025	19228
73	F	White	Former	2011	3	AC	22.4	2010	L3	17586	7847
63	M	White	Former	2011	1	AC	25.3	2011	L3	13172	21056
57	F	Asian	Never	2011	1	AC	19.8	2011	L3	14516	5484
74	M	White	Former	2011	1	AC	37.3	2010	L3	28080	45978
62	M	White	Former	2011	1	AC	29.5	2010	L3	19309	17975
74	F	White		2017	1	AC	28.4	2017	L3	20676	16512
68	F	White	Former	2011	3	AC	38.7	2011	L3	50422	13671
45	M	White	Former	2011	2	SCC	34.2	2011	L3	24850	25194
65	M	White	Former	2011	1	AC	25.8	2011	L3	12668	20823
65	M	White	Former	2011	2	AC	38.1	2011	L3	43396	34523
62	F	White	Current	2011	1	AC	30.2	2011	L3	20652	22990
76	M	White		2018	2	SCC	33	2018	L3	24550	42997
52	F	White	Former	2011	1	AC	17.8	2011	L3	3832	5268
64	F	White	Former	2011	1	AC	23.1	2011	L3	12071	13104
52	M	Black	Former	2011	3	AC	23.1	2011	L3	12468	9526
58	M	White	Current	2011	1	AC	25.0	2011	L3	12454	23465
65	M	White	Former	2011	1	AC	20.1	2011	L3	4662	8221
68	F	White	Current	2011	1	AC	17.4	2011	L3	4650	1865
75	F	White	Former	2011	1	AC	40.2	2011	L3	50509	19403
55	F	White	Former	2011	2	AC	27.8	2011	L3	22489	5496
69	M	White	Former	2011	2	SCC	30.0	2011	L3	32470	23941
61	F	White	Former	2011	2	Other	24.9	2011	L3	19046	9884
72	F	White	Former	2011	2	AC	28.3	2011	L3	25340	20587
49	F	Black	Current	2013		AC	27.2	2013	L3	30167	14544
61	M	White	Former	2011	2	AC	26.8	2011	L3	11271	30428
64	F	White	Current	2011	2	AC	25.1	2011	L3	16694	6848
60	F	White	Current	2011	3	Other	18.1	2011	L2	8045	6028
50	F	White	Current	2011	2	AC	19.6	2011	L3	10825	9258
74	F	White	Former	2011	1	SCC	40.3	2011	L3	33215	18760
67	M	White	Former	2011	1	SCC	38.2	2011	L3	51210	24333
54	F	Black	Never	2011	2	AC	35.8	2011	L3	36263	13319
75	M	White	Former	2011	1	Other	37.7	2011	L3	30100	45275
60	F	Black	Former	2011	1	AC	15.8	2011	L3	6248	5240
55	F	White	Current	2011	3	AC	32.4	2011	L3	31354	9674
34	F	White	Former	2011	1	AC	39.4	2011	L3	62388	12091
72	F	White		2018		Other	19.1	2018	L3	2128	3238
76	F	White	Former	2012	1	SCC	19.2	2011	L3	9631	10175

59	F	White	Current	2012	1	AC	23.5	2011	L3	19237	8566
58	F	White	Former	2011	1	SCC	29.3	2011	L3	35434	16126
61	F	White	Never	2011	1	AC	18.7	2011	L1	5232	6199
73	F	White	Former	2011	1	AC	36.2	2011	L3	37972	35934
77	F	White	Former	2011	1	AC	36.6	2011	L3	40206	36770
77	F	Asian	Former	2011	3	AC	18.4	2011	L3	7441	3512
68	F	White	Former	2011		AC	31.4	2011	L3	27771	7901
40	F	White	Former	2011	1	AC	28.9	2011	L3	34039	6481
56	F	White	Former	2011	1	AC	26.0	2011	L3	12325	3224
58	F	White	Former	2011	1	AC	30.7	2011	L3	30795	14476
74	M	White	Former	2011	2	AC	19.5	2011	L3	6237	9524
55	M	White	Former	2012	2	AC	24.6	2011	L3	11657	18055
68	F	White	Former	2011	1	AC	39.8	2011	L3	36693	27625
63	F	White	Former	2011	2	Other	27.4	2011	L3	13665	29177
65	M	White	Former	2011	3	AC	26.4	2011	L3	14384	18757
63	F	White		2018	2	Other	23.6	2018	L3	12715	12532
65	M	White	Former	2011	2	SCC	19.2	2011	L3	10007	9780
75	M	White	Former	2011	3	AC	29.2	2011	L3	10763	32217
71	F	White	Former	2012	2	AC	37.0	2011	L3	47358	28275
79	M	Black	Former	2011	1	SCC	27.1	2011	L3	20588	31021
81	M	White	Current	2011	1	SCC	22.8	2011	L3	6577	15682
80	F	White	Never	2011	3	AC	34.4	2011	L3	27176	36160
77	F	White	Former	2011	3	AC	22.9	2011	L3	21091	12195
55	M	White	Former	2011	2	AC	24.5	2011	L3	13933	16185
52	F	White	Former	2011	4	AC	25.3	2011	L3	24729	11706
62	F	White	Former	2011	1	AC	32.6	2011	L3	18287	21031
59	M	White	Former	2013	1	SCC	27.9	2013	L3	13988	26890
66	F	White	Never	2014	1	Other	23.5	2014	L3	16260	17643
88	F	White	Former	2012	1	SCC	20.9	2011	L3	13215	14934
55	M	White	Former	2012	1	SCC	29.7	2011	L3	19217	26587
77	M	White	Former	2012	2	SCC	28.4	2012	L3	16564	32816
66	M	White	Never	2012	1	AC	25.0	2012	L3	20163	18248
68	M	White	Former	2012	1	AC	24.1	2011	L3	15370	9878
83	M	White	Former	2012	2	AC	27.0	2012	L3	18825	33782
72	F	White	Former	2014	1	SCC	22.7	2014	L3	14482	7494
79	F	White	Former	2012	1	AC	35.1	2012	L3	41537	36987
65	F	White	Former	2012	1	SCC	25.9	2012	L3	22647	9075
74	F	White	Never	2012	1	AC	20.4	2012	L3	12762	3286
50	F	White	Former	2012	1	Other	26.6	2012	L3	26001	28255
74	M	Black	Former	2012	1	AC	32.0	2012	L3	16685	47135
81	M	White	Former	2014	2	SCC	24.8	2014	L3	21903	26113
70	M	White	Current	2012	1	SCC	25.4	2012	L3	16399	12051
68	M	Black	Former	2012	3	SCC	28.2	2012	L3	18236	16523
84	F	White	Current	2012	1	AC	25.7	2012	L3	12326	11373
79	M	White	Former	2012	1	AC	26.9	2012	L3	14600	24248
59	F	White	Former	2012	1	AC	23.1	2012	L3	18569	7377
49	F	White	Current	2012	1	AC	21.3	2012	L3	7781	1604
61	F	White	Former	2012	1	AC	26.2	2012	L3	17109	14215
49	F	White	Former	2012	2	SCC	29.2	2012	L3	21726	16151
65	M	White	Current	2012	2	AC	30.7	2012	L3	33086	29488
77	F	White	Former	2012	2	AC	34.6	2012	L3	27795	36308

64	F	White	Former	2012	1	SCC	20.4	2012	L3	6726	4481
56	F	White	Former	2012		SCC	34.8	2012	L3	28424	17962
60	F	White	Current	2012	1	SCC	27.2	2012	L3	25606	13334
73	F	White	Former	2012	1	SCC	21.1	2012	L3	17542	9128
68	F	White	Former	2012	1	AC	27.5	2012	L3	27344	19942
61	F	White	Former	2013	1	AC	28.2	2013	L3	12047	18385
69	F	White	Former	2012	1	AC	34.2	2012	L3	31714	18149
58	F	White	Former	2012	1	AC	24.4	2012	L3	12159	9537
65	F	White	Never	2012	1	AC	29.9	2012	L3	22230	20538
53	F	White	Never	2012	1	Other	36.9	2012	L3	28124	25667
69	F	White	Former	2014	1	AC	17.1	2014	L3	6308	3513
63	F	White	Current	2012	1	AC	16.3	2012	L3	6240	9087
64	M	White	Former	2012	2	SCC	29.6	2012	L3	22913	42249
64	F	White	Former	2012	2	Other	24.9	2012	L3	24385	10584
68	M	Black	Former	2013	1	AC	23.4	2013	L3	12995	8991
44	F	Asian	Former	2012	3	AC	23.2	2012	L3	13480	8418
52	F	White	Former	2012	1	AC	19.2	2012	L3	7465	4921
68	M	White	Former	2012	1	SCC	29.5	2012	L3	24832	32207
75	F	White	Former	2012	1	AC	23.5	2012	L3	24378	10779
66	M	White	Former	2012	1	AC	28.5	2012	L3	17932	18653
67	M	Black	Former	2012	1	AC	33.2	2012	L3	37118	33708
67	F	White	Former	2012	1	AC	25.0	2012	L3	19366	8238
69	F	White	Former	2012		Other	22.4	2012	L3	15615	13287
51	M	White	Former	2012	1	Other	25.6	2012	L3	17044	14222
53	M	White	Former	2012	1	AC	27.4	2012	L3	14168	24877
71	F	White	Former	2012	1	AC	30.9	2012	L3	31588	26379
68	F	White	Former	2012	3	AC	36.9	2012	L3	48332	10816
77	M	White	Former	2012	1	AC	35.5	2012	L3	26396	31049
74	M	White	Never	2012	1	AC	29.5	2012	L3	25786	35278
66	F	White	Former	2012	1	AC	24.5	2012	L3	24022	10347
68	F	White	Former	2012	2	AC	22.3	2012	L3	11538	8565
51	F	White	Former	2012	1	AC	44.1	2012	L3	49424	17548
73	F	White	Current	2012	2	AC	22.0	2012	L3	15221	9317
72	M	Asian	Former	2014	2	AC	24.5	2014	L3	12993	14819
63	F	White	Former	2012	2	SCC	28.5	2012	L3	22351	23610
61	F	White	Current	2012	1	SCC	28.6	2012	L3	18324	20408
70	M	White	Former	2012	1	SCC	26.7	2012	L3	20960	22900
67	M	White	Never	2012	2	AC	28.3	2012	L3	22623	27425
57	M	White	Former	2012	2	Other	22.0	2012	L3	8319	7062
54	M	Black	Current	2012	1	AC	30.7	2012	L3	23014	17046
65	F	White	Never	2012	1	AC	25.4	2012	L3	21138	14402
71	M	White	Former	2012	1	AC	25.8		L3	7236	36155
69	M	White	Former	2012	3	AC	30.9	2012	L3	29143	44810
65	F	White	Former	2015		AC	29.9	2015	L3	22003	20121
73	M	White	Former	2013	2	SCC	28.4	2013	L3	18682	34038
61	F	White	Current	2012	3	AC	32.4	2012	L3	40596	26055
63	M	White	Former	2012	1	AC	42.0	2012	L3	39176	38533
59	F	White	Former	2012	1	AC	26.0	2012	L3	27593	9823
70	M	White	Current	2012	2	SCC	25.0	2012	L3	14071	13755
56	M	White	Current	2012	1	SCC	28.1	2012	L3	20635	21872
54	M	White	Former	2013	1	SCC	18.9	2013	L3	7089	5203

51	F	White	Current	2013	1	AC	21.7	2013	L3	15692	10672
67	M	White	Former	2012	1	Other	36.7	2012	L3	27643	37577
72	F	White	Former	2012	1	AC	25.1	2012	L3	24772	13012
65	F	White	Former	2012	1	AC	26.1	2012	L3	22240	9643
78	M	White	Current	2012	1	SCC	28.7	2012	L3	15008	21824
61	F	White	Current	2015	1	SCC	28.5	2015	L3	34951	14175
72	M	White	Current	2012	2	SCC	19.8	2012	L3	10513	6084
63	M	Black	Current	2012	1	AC	19.2	2012	L3	3804	4572
58	F	White	Current	2012	1	AC	33.5	2012	L3	27328	20278
55	M	White	Current	2012	1	AC	27.5	2012	L3	14198	16257
62	F	White	Current	2012	1	AC	19.2	2012	L3	13734	3015
67	F	Black	Former	2012	1	AC	28.8	2012	L3	13774	12671
80	F	White	Former	2013	1	AC	28.6	2012	L2	22962	19582
81	M	White	Former	2012	2	Other	25.0	2012	L3	19795	28249
77	F	White	Current	2013	2	AC	21.7	2012	L3	11584	5463
62	M	White	Former	2013	2	AC	34.4	2012	L3	16465	33201
69	M	White	Former	2012	1	AC	26.3	2012	L3	13407	23435
58	F	Black	Former	2013	3	AC	24.1	2013	L3	15203	4804
71	M	White	Former	2013	1	AC	34.8	2012	L3	26997	43794
68	M	White	Former	2013	1	AC	29.0	2013	L3	15459	30837
67	F	White	Former	2014	1	AC	29.9	2014	L3	28111	11885
66	F	White	Current	2013	1	SCC	19.1	2013	L3	6672	2907
68	F	Black	Current	2013	1	AC	43.0	2012	L3	50862	29606
59	F	Other	Former	2013	2	AC	28.9	2013	L3	21220	23795
72	F	White		2018		AC	32.7	2018	L1	17349	16876
60	F	White	Former	2012	1	AC	30.0	2012	L3	34380	13117
71	M	White	Current	2013	2	SCC	25.9	2013	L3	19624	26482
72	F	White		2018		AC	25.2	2018	L2	13663	11872
55	F	White	Former	2013	1	SCC	27.8	2013	L3	20337	13295
75	M	White	Current	2013	2	SCC	36.3	2013	L1	26304	25103
70	M	White	Former	2013	3	AC	30.1	2013	L3	26331	25427
73	M	White	Former	2013	2	Other	31.2	2013	L3	36110	29885
64	F	White	Current	2013	2	AC	25.6	2013	L3	22551	22577
69	M	Asian	Former	2013	1	AC	25.5	2013	L3	10924	16451
44	M	White	Former	2013	1	AC	23.2	2013	L3	16669	16296
68	M	White	Current	2013	1	AC	27.6	2013	L3	17187	18415
80	F	White	Former	2013	3	SCC	28.9	2013	L3	24313	12261
87	M	White	Former	2013	1	SCC	25.1	2013	L3	12500	18559
65	M	White	Current	2013	1	SCC	27.9	2013	L3	14310	20917
74	M	White	Former	2013	2	SCC	27.4	2013	L3	14653	39431
58	F	White	Current	2013	1	AC	22.1	2013	L3	19395	7393
77	F	White	Former	2013	3	SCC	23.9	2013	L3	20003	11409
52	F	White	Current	2013	1	AC	23.0	2013	L3	21567	5303
64	M	White	Former	2013	2	SCC	33.2	2013	L3	27381	40952
68	M	White	Current	2013	2	SCC	30.4	2013	L3	22275	33977
66	M	Asian	Former	2013	1	AC	27.2	2013	L3	22582	35702
68	M	White	Former	2013	2	SCC	20.2	2013	L3	5868	4771
87	M	White	Former	2013	1	AC	27.5	2013	L3	15073	32701
69	F	White	Former	2013	1	SCC	25.3	2013	L3	30357	13574
56	F	White	Former	2013	1	AC	26.7	2013	L3	30569	11845
72	M	Asian	Never	2014	3	AC	18.6	2014	L2	7375	10523

76	F	White	Former	2013	1	SCC	30.5	2013	L3	25145	16307
57	F	White	Former	2013	2	AC	40.0	2013	L3	45717	26233
76	M	White	Former	2013	1	AC	34.2	2013	L3	26398	57091
48	F	Black	Current	2013	2	AC	24.2	2013	L3	19364	15346
55	F	White	Former	2013	1	AC	34.6	2013	L3	32601	23110
74	F	White	Former	2015		AC	27.3	2015	L3	19547	12198
62	M	White		2018	4	AC	31.4	2018	L3	32892	19714
65	M	White	Former	2013	1	SCC	28.8	2013	L3	19727	24112
76	F	White	Former	2013	2	SCC	34.2	2013	L3	30311	22332
71	F	White	Never	2013	1	Other	38.0	2013	L3	61989	29692
58	F	White	Former	2013	1	AC	39.8	2013	L3	46503	35018
53	F	White	Former	2013	1	AC	35.5	2013	L3	30479	20223
57	F	White	Former	2013	1	AC	27.4	2013	L3	22446	16071
76	F	White	Former	2013	2	AC	38.1	2013	L3	16691	37054
71	F	Black	Never	2013	1	AC	27.8	2013	L3	32366	16203
82	M	White	Former	2013	2	SCC	24.9	2013	L3	20719	21476
78	M	White	Former	2014	2	AC	31.6	2014	L3	17406	62400
73	F	White	Never	2013	1	AC	29.0	2013	L3	18781	24215
81	M	White	Never	2014	2	AC	26.3	2014	L3	15506	18459
44	F	White	Current	2013	3	AC	15.7	2013	L3	4374	3790
72	M	White	Former	2013	3	AC	29.0	2013	L3	19854	40560
68	F	White	Current	2013	1	SCC	29.2	2013	L3	33381	20149
76	M	White	Former	2013		SCC	28.9	2013	L3	30416	34460
70	M	White	Former	2013	3	SCC	26.7	2013	L3	21245	17480
79	F	White	Former	2013	1	AC	21.8	2013	L3	9826	12099
47	F	White	Current	2013	1	Other	39.4	2013	L3	53719	19738
70	F	White	Former	2014	4	AC	36.0	2014	L1	27110	12693
77	M	White	Former	2013	1	AC	27.7	2013	L3	12502	26110
86	M	White	Former	2013	2	SCC	23.2	2013	L3	9424	17338
57	F	White	Current	2013	1	AC	20.2	2013	L3	9116	3667
66	F	White	Former	2013	4	Other	26.1	2013	L3	27805	18460
69	M	Other	Never	2013	1	AC	29.8	2013	L3	31951	32637
69	M	White	Current	2013	3	AC	25.4	2013	L3	13691	11546
47	F	Black	Former	2014	1	AC	24.4	2014	L2	15117	1666
75	F	White	Former	2013	1	AC	23.0	2013	L3	13906	13463
58	M	Black	Former	2013	1	AC	32.2	2013	L3	30477	19521
70	F	White	Former	2013	3	SCC	24.9	2013	L3	17189	15390
79	M	White	Former	2013	1	Other	26.6	2013	L3	18452	30030
54	F	White	Current	2013	1	AC	19.3	2013	L3	11133	4358
73	M	White	Former	2013	1	SCC	21.1	2013	L3	10460	15568
66	M	White	Former	2013	2	SCC	30.4	2013	L3	17419	34936
65	F	White	Former	2013	2	SCC	22.9	2013	L3	18418	3562
77	F	White	Current	2015	1	AC	27.7	2015	L3	21936	9642
70	M	White	Former	2013	1	SCC	36.1	2013	L3	30088	57733
77	M	White	Former	2013	1	SCC	26.5	2013	L3	10850	26368
67	M	White	Current	2013	1	SCC	23.7	2013	L3	13503	13077
55	F	White	Current	2013	1	SCC	19.3	2013	L3	7897	3637
76	M	White	Former	2013	2	AC	21.3	2013	L3	14695	15251
70	M	White	Former	2014	1	SCC	28.1	2014	L3	15189	34383
56	M	Black	Current	2013	2	SCC	25.2	2013	L3	4324	2568
60	F	White	Former	2014	1	AC	28.4	2013	L3	24104	12340

77	M	White	Former	2013	1	AC	30.3	2013	L3	28961	41471
82	M	White	Former	2014	1	AC	25.3	2013	L3	13587	23345
55	F	White	Never	2014	1	AC	33.6	2013	L3	44567	15964
82	F	White	Former	2014	1	AC	24.4	2014	L3	12077	14762
84	M	Black	Former	2014	2	SCC	18.6	2014	L3	21075	10392
78	F	White	Former	2014	1	Other	29.2	2014	L3	21865	17858
60	M	White	Former	2013	1	AC	26.1	2013	L3	11893	25881
79	M	White	Former	2013	1	SCC	34.5	2013	L3	21781	48682
66	F	White	Former	2014	1	SCC	35.0	2013	L3	28201	34908
61	M	White	Former	2014	2	AC	27.3	2013	L3	14729	32731
74	F	Asian	Never	2013	1	AC	19.9	2013	L3	11636	12791
59	F	White	Current	2014	2	SCC	27.9	2013	L3	38191	14537
65	M	White	Former	2014	1	AC	24.1	2013	L3	7203	6913
65	F	White	Never	2014	1	AC	18.1	2013	L3	11794	7211
64	F	White	Current	2014	1	Other	30.6	2013	L3	27321	17958
78	F	White	Former	2014	3	AC	22.3	2013	L3	19783	8653
70	F	White	Current	2014	2	SCC	22.4	2013	L3	18446	12181
75	M	White	Former	2014	1	AC	33.1	2014	L3	28700	50708
56	M	White	Former	2014	1	AC	29.1	2014	L3	20230	18062
73	M	White	Current	2014	1	SCC	34.2	2013	L3	28867	29477
57	F	White	Former	2014	3	Other	24.4	2013	L3	27395	13443
53	M	White	Former	2014	1	SCC	34.5	2014	L3	42808	33216
69	F	White		2018	4	AC	21.1	2018	L3	14792	6389
71	F	White	Former	2014	1	AC	29.1	2014	L3	34669	12808
79	M	White	Former	2014	1	SCC	28.9		L3	13655	15946
71	M	White	Current	2014	2	Other	24.9	2013	L3	10055	18366
64	M	White	Former	2014	2	SCC	22.6	2014	L3	7267	8158
61	M	White	Former	2015		AC	26.6	2015	L3	12453	30245
46	F	White		2017		AC	17.6	2017	L3	3402	990
63	F	Black		2018	4	AC	18.6	2018	L3	6943	3418
71	M	White	Current	2014	2	SCC	25.0	2014	L3	24427	12448
81	F	Black	Former	2014	1	AC	28.4	2014	L3	19649	12349
67	F	White	Former	2014	1	AC	26.3	2014	L3	24026	15199
73	M	White	Former	2014	2	SCC	26.1	2014	L3	16097	13420
59	F	White	Never	2014	1	Other	29.4	2014	L3	33074	25192
80	M	White	Former	2014	1	AC	29.8	2014	L3	26152	30084
64	F	Other	Former	2014	1	AC	27.0	2014	L3	20220	9779
73	M	White	Former	2014	1	SCC	26.3	2014	L3	22503	24089
59	F	White	Current	2014	1	AC	26.8	2014	L3	20948	9492
64	M	White	Former	2017	2	SCC	35.8	2016	L3	25152	40519
76	F	White	Former	2014	2	SCC	23.1	2014	L3	12200	10353
77	F	White	Former	2015	1	AC	25.8	2014	L3	23651	8748
72	M	White	Former	2014	2	AC	33.6	2014	L3	12056	49201
71	F	Black	Former	2014	2	SCC	26.3	2014	L3	29639	16046
87	F	White	Never	2014	1	AC	27.0	2014	L3	17160	11050
79	F	White	Current	2014	1	AC	30.9	2014	L3	33552	19821
66	F	White	Former	2014	1	AC	22.1	2014	L3	18991	4320
66	F	White	Former	2014	1	AC	32.1	2014	L3	34315	19183
55	M	White	Current	2014	2	AC	26.2	2014	L3	11471	26098
78	M	White	Former	2014	1	AC	36.3	2014	L3	25827	54160
74	M	White	Current	2014	1	Other	21.9	2014	L3	11715	10123

80	M	White	Former	2014	3	SCC	35.2	2014	L3	23026	22216
67	M	White	Former	2014	1	SCC	33.9	2014	L3	24160	46144
62	F	White	Current	2014		SCC	23.9	2014	L3	21740	10199
60	M	White	Former	2014	1	AC	34.24	2014	L3	26010	39889
75	M	White	Former	2014	2	AC	25.64	2014	L3	17808	22066
71	F	White	Current	2014	1	AC	18.12	2014	L3	12177	3435
57	M	White	Former	2014	1	AC	27.92	2014	L3	19011	23740
79	F	Other	Former	2014	2	AC	30.36	2014	L3	32840	17787
73	F	White	Current	2015	1	AC	31.03	2015	L3	28685	15850
67	F	White	Former	2014	1	SCC	33.43	2014	L3	33696	20160
66	F	White	Never	2014	1	AC	37.93	2014	L3	41947	23636
71	F	White	Former	2014	1	SCC	23.67	2014	L3	25101	27412
75	F	White	Former	2014	3	AC	31.04	2014	L2	17026	19686
82	M	White	Former	2014	2	SCC	28.15	2014	L3	16387	37792
66	F	White	Former	2014	2	Other	30.72	2014	L3	25483	24283
67	F	Other	Never	2014		AC	26.32	2014	L3	15862	18617
87	M	White	Former	2014	2	SCC	23.96	2014	L3	14529	27450
60	M	White		2018		AC	32.9	2018	L3	28526	8398
66	M	White	Former	2014	2	SCC	32.52	2014	L3	21532	25310
79	F	White	Current	2015	2	AC	23.54	2015	L3	11768	9942
52	F	White	Current	2014	1	SCC	19.06	2014	L3	8335	4369
82	F	White	Former	2014	1	AC	30.71	2014	L3	34631	21836
56	F	White		2018	3	AC	27	2018	L3	26170	20757
82	F	White	Current	2014	1	SCC	26.75	2014	L3	27588	19645
70	F	White	Former	2015	2	SCC	43.91	2014	L3	56870	31603
79	F	White		2016	1	AC	35.4	2016	L1	37836	17347
71	F	White	Former	2015	3	Other	40.08	2014	L3	39289	20363
73	M	White	Former	2015		AC	26.92	2014	L3	15070	26283
65	F	White	Former	2014	1	AC	30.97	2014	L1	18507	10644
73	F	White	Former	2015	2	AC	23.99	2014	L2	11778	8366
63	F	Black		2018	3	AC	35.6	2018	L3	37671	22701
75	M		Former	2015	1	AC	36.85	2015	L3	32157	51708
65	M	White	Former	2015	4	AC	31.23	2015	L3	22715	38412
77	M	White	Former	2014	2	AC	32.9	2014	L3	21312	51487
74	F	White	Former	2015	2	AC	30.76	2014	L1	20907	16481
74	M		Former	2015	3	SCC	23.77	2014	L3	12043	19808
84	F	White	Former	2015		Other	30.83	2014	L3	32207	31002
54	F	White	Current	2015	2	AC	20.39	2014	L3	21004	4684
54	F	White	Current	2015	1	AC	30.06	2014	L3	34759	9852
78	F	White	Current	2015	1	SCC	22.03	2015	L3	12357	4547
77	F	White	Current	2015	1	AC	24.55	2015	L3	21290	21116
67	F	White	Former	2015	1	AC	36.32	2015	L3	39014	23344
74	M	White	Former	2015	1	AC	26.55	2015	L3	16545	28453
58	F	White	Former	2015	1	SCC	34.4	2014	L3	40656	30380
78	F	White	Former	2015	2	AC	32	2014	L3	41931	30637
70	F	White	Former	2015	2	AC	30.44	2014	L3	27689	34770
83	F	White	Former	2015	1	AC	22.54	2015	L3	16986	5960
72	M	White	Former	2015	1	AC	31.35	2015	L3	26390	32727
66	M	White		2018	4	AC	27.4	2018	L3	15418	29851
70	M	White	Current	2015	1	SCC	39.58	2015	L3	49775	48466
54	F	White	Former	2015	1	AC	22.74	2015	L3	21829	10277

76	M	White	Former	2015	2	SCC	34.76	2015	L3	13972	37988
63	F	White	Former	2015	1	AC	27.39	2015	L3	22101	17601
42	F	White	Former	2015	2	AC	36.07	2015	L3	47205	7847
77	M	White	Never	2015	2	AC	24.36	2015	L3	9469	11421
68	M	White	Former	2015	1	AC	27.76	2015	L3	14377	19766
61	M	White	Current	2015	1	AC	23.22	2015	L3	10757	6141
59	F	White	Current	2015	1	AC	27.51	2015	L3	27296	22517
59	F	White	Former	2015	1	AC	29.78	2015	L3	19328	10012
53	M	White	Never	2015	3	AC	30.71	2015	L3	24036	25737
69	F	White	Former	2015	2	SCC	25.8	2015	L3	18698	20764
74	M	White	Former	2017	2	SCC	31.3	2016	L3	13778	35033
75	M	White	Former	2015	1	SCC	28.02	2015	L3	18809	36911
65	F	White	Former	2015	1	AC	31.76	2015	L3	32302	24696
72	F	White		2018	4	AC	31.7	2018	L1	27314	19403
68	M	White	Former	2015	1	AC	22.79	2015	L3	10140	4186
67	F	White	Never	2015	1	Other	33.76	2015	L3	28027	15416
69	F	White		2016	3	AC	46.5	2016	L3	62941	31047
61	F	White	Current	2015	1	SCC	20.54	2015	L3	14605	14171
54	M	White		2015		SCC	26.8	2015	L3	17426	20340
72	F	White	Former	2015	3	AC	35.78	2015	L3	33112	28213
78	M	White	Current	2015	2	AC	27.31	2015	L3	21458	22717
66	F	White	Current	2015	2	AC	23.02	2015	L3	20534	9934
60	M	White	Never	2015	1	AC	27.3	2015	L3	19526	26103
47	F	White	Former	2015	1	AC	24.37	2015	L3	17357	9572
71	F	White	Former	2015	1	AC	28.76	2015	L3	24917	18317
67	M	White	Never	2015	2	SCC	22.86	2015	L3	11086	7171
84	F	White	Former	2015	1	AC	29.63	2015	L3	21278	21040
65	F	White	Former	2015	2	AC	31.82	2015	L3	38820	37204
86	F	White	Former	2015	1	SCC	25.97	2015	L3	28482	16394
49	M	White		2017		AC	18.7	2017	L2	4506	2872
69	F	White	Former	2015	1	AC	33.96	2015	L3	29013	26714
79	F	White	Former	2015	1	SCC	29.47	2015	L3	22542	11115
71	F	White	Former	2015	1	SCC	19.61	2015	L3	10631	9474
75	F	White	Former	2015	2	SCC	25.74	2015	L3	16394	10082
46	M	White	Current	2015	2	SCC	36.21	2015	L3	35753	27433
76	F	White	Never	2015	2	AC	27.92	2015	L3	21898	18146
67	F	White	Former	2015	2	AC	35.81	2015	L3	43028	35204
46	F	White	Former	2015	1	AC	24.65	2015	L3	18789	6193
69	M	White	Former	2015	2	AC	36.17	2015	L3	51943	39484
73	F	White	Never	2015	3	SCC	33.05	2015	L3	39234	27363
71	F	White	Former	2015	1	Other	28.28	2015	L3	21000	22216
65	M	White	Former	2015	1	AC	27.34	2015	L3	12031	19152
63	F	White	Never	2015	1	Other	37.29	2015	L3	49281	21373
73	M	White	Former	2015	2	SCC	29.26	2015	L3	22168	35977
29	F	White	Former	2015	2	Other	24.04	2015	L1	4813	2195
73	M	White	Current	2015	1	AC	23.89	2015	L3	9920	12807
68	F	White	Never	2015	1	AC	27.47	2015	L3	24547	9852
73	M	White	Former	2015	1	SCC	27.62	2015	L3	12811	24403
65	M	White	Former	2015	1	AC	18.36	2015	L3	5566	9604
63	M	White	Current	2015	1	SCC	29.89	2015	L3	16318	30354
80	F	White	Former	2015	3	Other	17.8	2015	L3	3418	4010

67	F	White		2015		AC	19.8	2015	L3	5142	3854
62	F	White	Current	2015	1	AC	24.95	2015	L3	23067	11767
77	M	White	Former	2015	1	SCC	26.85	2015	L3	22887	36468
69	F	White	Former	2015	1	AC	25.62	2015	L3	14397	11252
86	F	Black	Former	2015	1	SCC	30.35	2015	L3	29685	10854
70	M	White	Current	2015	1	AC	34.61	2015	L3	37702	37884
78	M	White	Former	2015	1	AC	25.21	2015	L3	21918	16432
76	F	White	Former	2015	3	AC	21.14	2015	L3	8737	6351
38	F	White	Current	2015	3	Other	38.16	2015	L3	53190	20330
83	F	White	Former	2015	2	AC	31.38	2015	L3	38179	17919
59	F	White	Former	2015	1	AC	26.06	2015	L3	37454	9537
62	M	White	Former	2015	1	AC	26.74	2015	L3	13941	22035
64	F	White	Former	2015	1	AC	36.52	2015	L3	31043	31997
66	M	White	Former	2015	1	AC	26.06	2015	L3	14815	27413
80	F	White	Never	2015	2	AC	22.44	2015	L3	20719	13914
70	M	White	Former	2015	1	AC	41.99	2015	L3	34384	69879
59	F	White	Former	2015	1	AC	25.85	2015	L3	23832	10705
68	M	White	Former	2015	1	Other	28.28	2015	L3	15716	21285
49	F	White	Current	2015	1	AC	23.17	2015	L3	16233	9479
56	M		Current	2015	2	SCC	31.96	2015	L3	25997	26335
66	F	White	Never	2015	1	AC	29.37	2015	L3	22244	13829
72	F	White	Current	2015	2	SCC	23.85	2015	L3	22411	5231
56	M	White		2017		Other	27.4	2017	L3	16207	24511
54	F		Former	2015	1	AC	38.25	2015	L3	52808	22337
70	F	White	Former	2015		SCC	36.71	2015	L3	36734	25086
69	F	White	Former	2015	1	SCC	44.89	2015	L3	55332	26000
64	F	White	Current	2015	1	AC	29.6	2015	L3	22465	33238
79	M	White		2017	3	AC	39.9	2017	L3	67201	29611
66	M	Black		2017		SCC	22.1	2017	L3	15946	14581
70	F	White	Former	2015	1	AC	29.53	2015	L3	31426	10465
55	M	White	Former	2015	1	AC	26.16	2015	L3	21571	21266
64	F	White		2018		AC	23.6	2018	L3	20720	10761
65	F	White	Former	2015	1	SCC	36.18	2015	L3	43905	19960
53	F	White		2018		AC	30.6	2017	L3	18919	14495
57	F	White	Current	2015		AC	20.46	2015	L3	10221	2703
59	F	Black	Current	2015	1	Other	25.03	2015	L3	31094	13515
70	F	White		2018	4	AC	25.6	2018	L3	19775	10955
56	M	Asian		2017		AC	23.3	2017	L1	4421	6821
50	M	White	Current	2016	2	AC	18.9	2016	L3	2749	3584
65	F	White		2016	2	AC	23.8	2016	L3	21443	26537
73	M	White		2018		SCC	32.2	2018	L3	30122	45868
77	M	White		2018	2	AC	29.6	2018	L3	10490	51959
73	F	White	Former	2017	2	AC	20.7	2017	L3	32308	20321
67	M	White		2016		SCC	26	2016	L3	14001	35294
47	F	White	Former	2016	2	AC	30.2	2016	L3	40422	10873
75	M	White	Current	2016	2	SCC	28.4	2016	L3	14780	34077
60	F	White		2016	1	AC	23	2016	L3	19054	4659
66	F	Other		2018	4	AC	20.5	2018	L2	11786	4686
61	M	White		2018	4	AC	28.3	2018	L3	16320	27735
79	F	Black		2016		AC	26	2016	L3	24596	21513
68	M	White		2016		SCC	30.5	2016	L2	14207	44286

55	F	White	Current	2019	3	AC	22.2	2019	L1	8845	7420
65	M	White	Former	2016	2	SCC	31.2	2016	L3	40313	23350
74	F	White		2017		AC	31.2	2017	L2	24161	10181
65	M	Other		2018	4	AC	24.2	2018	L3	15268	20354
70	M	White	Former	2016	2	AC	30.5	2016	L3	17728	27752
49	M	White	Former	2016	2	SCC	19	2016	L1	2995	4082
61	M	White	Former	2017	3	AC	26.5	2017	L3	14049	13880
61	F	White	Current	2016	2	AC	17	2016	L3	3803	1038
64	F	White		2016		SCC	25.2	2016	L3	14306	7001
58	M	White	Current	2016	3	AC	26	2016	L3	15544	13934
43	F	White	Never	2016	2	Other	24.8	2016	L3	20946	5484
47	M	White	Current	2016	4	AC	27.4	2016	L3	13076	17879
73	F	White	Former	2017	2	Other	31.6	2017	L3	30432	14082
62	M	White	Current	2016	2	AC	22.7	2016	L3	5754	24790
74	M	White		2016		AC	29.4	2016	L3	14127	33968
70	F	White	Former	2017	3	AC	32.8	2016	L1	27146	17833
62	M	White	Current	2017	2	AC	26.6	2016	L3	16545	22436
63	F	White	Former	2017	3	AC	22.3	2017	L3	22108	7799
66	M	White		2017		AC	30.3	2017	L3	30884	20698
72	F	White		2017	4	AC	28.4	2017	L3	12516	15290
74	F	White	Former	2017	2	SCC	25.9	2017	L3	33409	22725
81	M	White		2017		SCC	32.4	2017	L3	29019	32966
56	M	White		2017		SCC	31.7	2017	L3	13960	34776
65	F	White	Current	2017	2	SCC	22.8	2017	L3	19393	12049
60	M	White		2018		Other	21.6	2018	L3	16263	8564
89	M	White	Current	2017	3	SCC	26.1	2017	L3	16434	21146
75	M	White	Current	2019	4	SCC	15.7	2019	L3	3354	5311
57	F	White	Current	2017	2	AC	24	2017	L3	14751	9902
86	F	White		2017		AC	28.1	2017	L3	34088	25476
66	M	White	Former	2017	3	SCC	24.1	2017	L3	11859	17810
68	F	White	Former	2017	3	AC	26.5	2017	L3	20949	20710
63	F	White	Former	2017		AC	34.3	2017	L3	24723	22570
75	F	White		2017		AC	21.1	2017	L3	8326	10357
68	F	White		2017		AC	28.2	2017	L3	25274	14875
60	F	White	Current	2017	2	SCC	30.4	2017	L3	27754	14734
54	F	White	Former	2017	1	AC	31	2017	L3	44783	15147
54	F	White		2018		AC	29.7	2018	L2	23198	13247
70	M	White	Former	2017	2	Other	31.9	2017	L3	17249	46212
62	F	White	Former	2019	3	AC	27.4	2019	L3	34730	19224
61	M	White	Current	2017	4	Other	38.2	2017	L3	28564	40710
84	F	White	Former	2017	2	Other	24.4	2017	L3	7767	8359
56	M	White		2017	1	Other	20.5	2017	L3	5484	4173
76	F	White		2017		SCC	29.9	2017	L3	42197	17272
66	M	White	Current	2017	4	AC	20.7	2017	L3	3377	7331
55	M	White		2017	4	AC	26.2	2017	L3	12689	18619
73	F	Black		2017		AC	23	2017	L3	25222	5945
62	M	White		2017		AC	23.8	2017	L3	11771	10681
64	F	White		2017	4	AC	31.3	2018	L3	26751	16074
73	M	White		2018		SCC	29.2	2018	L3	17775	28369
56	F	White	Current	2017	2	AC	24.3	2017	L3	14771	4404
55	M	White		2017		AC	31	2017	L3	18281	38945

59	F	White		2017	4	AC	23.7	2017	L3	21658	16743
59	M	White		2017		Other	24.3	2017	L3	13360	26968
73	M	White	Former	2017	3	AC	27.1	2017	L3	24058	24154
73	F	White		2017		AC	30.5	2017	L3	26470	22697
69	M	Asian		2017		AC	27.1	2017	L3	15814	24598
76	M	White	Never	2017	3	SCC	22.6	2017	L3	7074	11595
56	M	White		2017		AC	32.6	2017	L3	18728	35099
71	M	White	Former	2017	3	AC	27.4	2017	L3	12648	26497
49	M	Asian		2017	4	AC	30.9	2017	L3	15940	39196
69	M	Other		2017		Other	25.5	2017	L3	14379	20632
53	M	White		2018	1	AC	29.6	2018	L3	15807	20664
55	F	White	Current	2017	4	AC	22.4	2017	L3	14799	10264
78	F	White		2017	2	AC	27.5	2017	L3	22566	23780
63	M	White		2017		AC	33.9	2017	L3	37233	24753
77	M	Black		2018		SCC	21.5	2018	L3	10108	20348
66	M	White		2018	3	AC	30.1	2018	L3	31723	30111
64	F	White		2017		SCC	17.2	2017	L3	4206	3126
65	F	White		2017	4	AC	23.7	2017	L3	16921	10266
52	F	White		2017		AC	25.8	2017	L3	24819	13951
84	M	White		2017		Other	28.2	2017	L3	11771	31642
69	M	White		2017		AC	31.8	2017	L3	21094	30881
74	M	White		2017	3	SCC	31	2017	L3	20867	41171
52	F	White		2017	4	Other	20.9	2017	L2	9994	6016
61	F	White		2017	4	AC	30.9	2017	L3	27191	27279
74	F	White		2017	2	AC	24.7	2017	L3	21022	14795
63	M	White		2017		AC	19.6	2017	L3	8357	5498
74	M	White	Former	2018	2	SCC	23.1	2018	L3	7530	9163
55	F	White		2017	4	AC	23.3	2017	L3	17139	7806
26	F	White		2017		AC	35.7	2017	L3	48637	13874
64	M	White	Former	2018	4	AC	30.3	2017	L3	28466	39016
75	F	White		2017		AC	20.6	2017	L3	10672	14501
78	F	White		2017		Other	30.4	2017	L3	24291	18833
72	F	White		2017		Other	25.7	2017	L3	27445	21074
78	M	White		2017	3	AC	24.9	2017	L3	21965	14952
75	M	White		2018	4	AC	19.1	2018	L3	6326	6647
76	M	White		2018		SCC	35.5	2018	L3	15865	57351
61	F	Black		2018		AC	20.2	2018	L3	14991	11884
67	M	White		2018		SCC	22	2018	L3	6430	11561
64	M	White		2018	4	AC	30.8	2018	L3	23656	25404
86	F	White		2018	4	AC	21.8	2018	L2	6729	5051
38	F	White		2017	4	AC	32.1	2017	L3	31867	15121
89	F	White		2017		AC	22.3	2017	L3	12668	10315
57	M	White		2017		AC	31.4	2017	L3	28215	20739
61	M	White	Current	2019	4	Other	31.9	2018	L3	14943	40570
84	M	White		2017		AC	25.8	2017	L3	13184	18672
84	F	White		2017	4	AC	20.1	2017	L3	4497	3239
53	F	White		2017	4	AC	27.5	2017	L3	30837	17435
85	F	White		2017	4	AC	23.5	2017	L3	9496	10612
74	F	Asian		2017		AC	14.5	2017	L3	4593	2555
58	F	White		2018		AC	32.1	2018	L3	39288	16150
63	F	White		2018	4	AC	24.6	2018	L3	16360	11124

64	M	White		2018		AC	26.7	2018	L3	12672	24537
72	F	White		2018	1	SCC	21.6	2018	L3	10978	6989
47	F	White		2017	4	AC	37.3	2018	L3	39931	18955
43	M	Black		2018	4	Other	19.5	2018	L3	3158	5408
71	M	White	Former	2018	1	AC	32.6	2018	L3	37251	37218
79	F	White		2018	4	AC	27.5	2018	L3	29835	13293
49	M	White		2018		SCC	20.6	2018	L3	3450	3129
50	F	White		2018		AC	33.6	2018	L3	48112	17323
65	M	White		2018	4	AC	26.1	2018	L3	16478	22329
58	M	White		2018	4	AC	22.2	2017	L3	9370	10943
61	M	White		2018		AC	22.4	2018	L3	3562	5232
64	F	White		2018	4	Other	37.3	2018	L3	48351	28658
58	F	Black		2018		AC	29	2018	L3	30336	8514
71	M	White		2018		SCC	34.5	2018	L3	30441	41829
61	M	White		2018		Other	29.4	2018	L3	24316	24705
54	F	White		2018	4	AC	32.5	2018	L3	41178	16180
81	M	White		2018		Other	31.1	2018	L2	11231	30004
69	F	White		2018		AC	31.3	2018	L3	40271	20435
53	F	Black		2018	4	AC	19.2	2018	L3	11331	7163
71	F	White		2018		AC	27.9	2018	L3	19195	15342
74	M	White		2018		AC	26.9	2018	L3	21727	35016
74	F	White		2018	4	AC	25.9	2018	L3	18032	16021
80	M	White		2018		AC	24.1	2018	L3	13439	11446
68	F	White	Former	2019		AC	34	2019	L3	40904	19026
45	M	White	Former	2019		Other	28.8	2018	L3	17294	24590
71	F	White		2018	4	AC	26.1	2018	L3	36996	19982
80	F	White		2018		AC	25.9	2018	L3	14024	12930
68	M	White		2018		Other	27.9	2018	L1	9296	32735
60	M	Black		2018		AC	16	2018	L3	2124	2508
56	F	White		2018	4	AC	30.5	2018	L3	17294	10221
69	M	White		2018	4	Other	27.7	2018	L3	16196	29805
59	F	White		2018		SCC	15.1	2018	L3	476	970
79	F	White		2018	4	AC	24.3	2018	L3	18379	12440
90	M	White		2018	3	AC	22	2018	L3	8563	20520
56	M	White		2018		Other	29	2018	L3	21579	29902
66	M	White		2018		SCC	25.1	2018	L3	14805	15198
64	M	White		2018	4	Other	26.5	2018	L3	20551	33282
57	M	White		2018		Other	26.2	2018	L3	17318	12357
80	F	White		2018	4	AC	29.4	2018	L3	17203	16269
81	M	White		2018	4	AC	20.4	2018	L3	9795	13507
68	F	White		2018		AC	28.2	2018	L3	23680	16352
55	M			2018	4	AC	19.3	2018	L3	5496	11073
79	F	Black		2018		Other	29.1	2018	L3	15312	10224
64	M	White		2018		AC	25.6	2018	L3	10405	17203
70	M	White		2018		AC	31.5	2018	L3	21200	39230
57	M	White	Current	2018	4	AC	22.1	2018	L3	6526	11723
49	F	White		2018	4	AC	25.2	2018	L3	21290	23532
64	F	White	Never	2018		Other	29.6	2018	L3	21716	17752
74	F	White	Current	2018	1	AC	38.1	2018	L3	3877	1756
65	M	White	Current	2019		Other	26.2	2019	L3	16005	24816
74	F	White	Current	2019	1	AC	26.6	2019	L1	27189	21040

67	M	White	Current	2018	3	Other	33.9	2018	L3	19971	34239
56	F	White	Current	2019		Other	32.5	2019	L3	30199	20343
81	M	White	Former	2019	3	SCC	28.9	2018	L3	23385	30889
67	M	White	Current	2019	2	SCC	36.8	2019	L1	19923	51046
54	F	White	Former	2019	3	AC	23.1	2019	L3	14921	5797
81	M	White	Former	2019	3	SCC	25.9	2019	L3	17263	24151
71	F	White	Current	2019	2	SCC	34.6	2019	L3	30713	36308
70	M	White	Former	2019	4	Other	38.9	2019	L1	11922	31568
51	F	White	Never	2019	1	AC	33	2019	L3	43410	20523
58	F	White	Current	2019		AC	30.1	2019	L3	31011	21539
68	F	White	Former	2019	1	AC	22.8	2019	L3	12249	10061
65	F	White	Current	2019	1	SCC	24.9	2019	L3	27327	12948
69	M	White	Current	2019	3	SCC	32.5	2019	L3	27655	25547
73	M	White	Former	2019	3	AC	30.8	2019	L3	18388	40856
63	F	White	Current	2019	2	AC	29.3	2019	L3	35297	23401
62	F	White	Current	2019	1	SCC	25.5	2019	L3	21588	13488
60	F	White	Former	2019	1	AC	33.1	2019	L3	50422	13264
73	F	White	Former	2019	2	SCC	24.2	2019	L3	28318	11361
48	M	White		2019	3	SCC	27.2	2019	L3	13375	16849
68	F	Other	Never	2019	1	AC	30.5	2019	L3	31310	20712
69	M	White	Former	2019	2	Other	30.6	2019	L3	18662	49508
59	F	White	Former	2019	1	Other	21.2	2019	L3	11420	4680
43	F	White	Former	2019	2	AC	27.1	2019	L3	25043	13472
74	M	White	Current	2019	1	AC	23	2019	L3	7398	11600
74	F	White	Former	2019	2	AC	28.9	2019	L3	24971	16376
73	M	White	Former	2019	1	AC	27.8	2019	L3	11800	36441
62	F	White	Former	2015	1	Other	36.89	2015	L3	35832	38632
66	M	White	Former	2012	3	SCC	25.92	2012	L3	19306	24276
64	M	White	Former	2020		Other	23.9	2020	L1	8701	13378
84	F	White	Former	2019	1	AC	14.7	2019	L3	3708	2863
62	M	White	Former	2020		Other	19.6	2020	L3	4045	3198
80	F	White	Former	2019	2	Other	19.8	2019	L3	5772	2335
62	F	White	Current	2019	4	SCC	18.4	2019	L3	7250	3589
58	F	Black	Current	2020	2	Other	20.4	2019	L3	8880	3820
71	F	White	Former	2020	1	Other	18	2019	L3	9762	3575
51	M	White	Former	2020	1	AC	26.5	2019	L3	8389	7376
57	M	White	Former	2020		SCC	22.1	2020	L3	8597	8275
75	M	White	Former	2020	4	Other	24	2020	L3	8692	13012
70	F	White	Former	2019	1	AC	22.9	2019	L3	18494	4459
47	M	White	Current	2020	1	AC	21.1	2019	L3	10217	13947
57	F	White	Never	2019	3	AC	23.8	2019	L3	20638	3553
63	M	Black	Current	2020	1	AC	24.6	2019	L3	10900	15296
83	M	White	Former	2019	2	AC	21.9	2019	L3	15921	10625
68	M	White	Former	2020		SCC	23.6	2020	L3	9752	16954
65	M	White	Current	2020	1	Other	19	2019	L3	10244	16473
65	M	White	Current	2019	3	AC	22.7	2019	L3	9972	17447
63	F	White	Former	2020	3	AC	23.5	2019	L3	14726	13810
71	M	White	Current	2019	2	Other	21.9	2019	L3	11910	16968
75	F	White	Former	2020	3	AC	21.2	2019	L3	21136	8187
64	M	White	Former	2020		Other	23	2020	L3	13765	16105
63	F	White	Former	2019	1	AC	23.8	2019	L3	16528	13581

79	F	White	Former	2020	3	AC	25.4	2020	L3	18237	13993
79	M	White	Former	2019	1	Other	21.9	2019	L3	13214	19565
72	M	White	Former	2019	1	AC	27.4	2019	L3	15101	18042
64	F	White	Former	2019	1	AC	20.8	2019	L3	22249	11343
66	F	Black	Former	2019	1	AC	33.7	2019	L3	24131	9558
74	F	White	Former	2019	3	AC	25.1	2019	L3	21655	13534
81	F	White	Former	2020	1	SCC	30.9	2019	L3	26673	9473
63	M	White	Current	2019	3	Other	27.2	2019	L3	18541	18461
78	F	White	Former	2020	1	AC	30.7	2019	L3	27389	10631
78	M	White	Former	2019		Other	26.5	2019	L3	23375	15492
85	M	White	Former	2020	3	Other	27.2	2020	L3	16328	23111
69	F	White	Never	2019	3	AC	26.9	2019	L3	22259	17653
60	M	White	Former	2019	2	AC	26.7	2019	L3	27005	13067
66	F	White	Current	2020	1	AC	26.1	2020	L3	26695	13486
53	F	White	Never	2020		AC	25.6	2020	L3	17495	24027
63	M	White	Former	2020	3	AC	29.2	2019	L3	21790	19868
60	F	White	Former	2019	1	AC	27.5	2019	L3	21344	21025
60	F	White	Current	2020	3	Other	29.1	2020	L3	28156	14888
63	F		Current	2020	4	AC	24.2	2020	L3	23895	19405
58	F	White	Former	2019	3	AC	27.7	2019	L3	31784	11898
56	M	White	Former	2019	3	Other	30.4	2019	L3	24477	19613
84	F	White	Former	2019	1	AC	29.3	2019	L3	24288	21415
76	M	White	Former	2019	1	SCC	28.4	2019	L3	12819	35556
67	M	White	Former	2019	1	AC	31.8	2019	L3	19380	31237
84	M	White	Former	2020	2	AC	29.9	2019	L3	10132	41372
59	M	White	Former	2020	1	SCC	29.8	2019	L3	15697	35895
67	M		Former	2020	1	AC	28.2	2019	L3	25899	26307
73	M	White	Former	2020	4	Other	30.5	2020	L3	26366	26532
64	F	White	Current	2020	1	AC	25.7	2020	L3	30996	22503
66	M	White	Former	2019	1	AC	27.1	2019	L3	14136	42744
61	F	White	Former	2019	1	AC	39.2	2019	L3	29218	29547
83	M	White	Former	2020	2	Other	28.8	2019	L3	24262	34872
74	F	White	Former	2019	3	AC	31.4	2019	L3	25960	34543
83	F	White	Former	2019	1	AC	29.8	2019	L3	33604	27837
82	M	White	Former	2020	1	AC	30.5	2019	L3	26419	38985
64	M	White	Current	2020		AC	38.3	2020	L3	22133	43640
57	F	White	Former	2019	4	AC	22.8	2019	L3	14147	3829

[a]Listed are abdominal-level body fat area measurements determined using ImageJ software and computerized tomography (CT) scans of 994 unique subjects with non-small cell lung cancer tumors, along with their other characteristics. Only incomplete information was available for some subjects. *AC*, adenocarcinoma; *F*, female; *L*, lumbar; *M*, male; *SCC*, squamous cell carcinoma.

Table S2. STROBE statement with items recommended for reports of cohort studies[a]

		Item	*Page*
Title and abstract	1	(*a*) Indicate the study's design with a commonly used term in the title or the abstract	1
		(*b*) Provide in the abstract an informative and balanced summary of what was done and what was found	1
Introduction			
Background/rationale	2	Explain the scientific background and rationale for the investigation being reported	1-2
Objectives	3	State specific objectives, including any prespecified hypotheses	2
Methods			
Study design	4	Present key elements of study design early in the paper	3
Setting	5	Describe the setting, locations, and relevant dates, including periods of recruitment, exposure, follow-up, and data collection	3
Participants	6	(*a*) Give the eligibility criteria, and the sources and methods of selection of participants. Describe methods of follow-up	3
		(*b*) For matched studies, give matching criteria and number of exposed and unexposed	NA
Variables	7	Clearly define all outcomes, exposures, predictors, potential confounders, and effect modifiers. Give diagnostic criteria, if applicable	3-4
Data sources/ measurement	8*	For each variable of interest, give sources of data and details of methods of assessment (measurement). Describe comparability of assessment methods if there is more than one group	4
Bias	9	Describe any efforts to address potential sources of bias	4-5
Study size	10	Explain how the study size was arrived at	3
Quantitative variables	11	Explain how quantitative variables were handled in the analyses. If applicable, describe which groupings were chosen and why	4-5
Statistical methods	12	(*a*) Describe all statistical methods, including those used to control for confounding	4-5
		(*b*) Describe any methods used to examine subgroups and interactions	4-5
		(*c*) Explain how missing data were addressed	NA
		(*d*) If applicable, explain how loss to follow-up was addressed	NA
		(*e*) Describe any sensitivity analyses	NA
Results			
Participants	13*	(a) Report numbers of individuals at each stage of study—eg numbers potentially eligible, examined for eligibility, confirmed eligible, included in the study, completing follow-up, and analysed	4-9
		(b) Give reasons for non-participation at each stage	NA
		(c) Consider use of a flow diagram	NA
Descriptive data	14*	(a) Give characteristics of study participants (eg demographic, clinical, social) and information on exposures and potential confounders	4, 7
		(b) Indicate number of participants with missing data for each variable of interest	18

		(c) Summarise follow-up time (eg, average and total amount)	NA
Outcome data	15*	Report numbers of outcome events or summary measures over time	NA
Main results	16	(a) Give unadjusted estimates and, if applicable, confounder-adjusted estimates and their precision (eg, 95% confidence interval). Make clear which confounders were adjusted for and why they were included	4-10
		(b) Report category boundaries when continuous variables were categorized	4-10
		(c) If relevant, consider translating estimates of relative risk into absolute risk for a meaningful time period	NA
Other analyses	17	Report other analyses done—eg analyses of subgroups and interactions, and sensitivity analyses	4-10
Discussion			
Key results	18	Summarise key results with reference to study objectives	10
Limitations	19	Discuss limitations of the study, taking into account sources of potential bias or imprecision. Discuss both direction and magnitude of any potential bias	10-11
Interpretation	20	Give a cautious overall interpretation of results considering objectives, limitations, multiplicity of analyses, results from similar studies, and other relevant evidence	10-11
Generalisability	21	Discuss the generalisability (external validity) of the study results	10-11
Other information			
Funding	22	Give the source of funding and the role of the funders for the present study and, if applicable, for the original study on which the present article is based	12

[a]Page numbers refer to the submitted manuscript document. *STROBE*, Strengthening the Reporting of Observational Studies in Epidemiology.

Table S3. Sex-specific characteristics of the examined population at the L3 vertebral level[a]

	Female *(N = 530)*	*Male* *(N = 417)*	*P value*[b]
Age at time of analyzed CT (years)	66 (59, 74)	68 (62, 74)	0.003
Race			0.800
White	476 (90.2%)	373 (90.8)	
Black	40 (7.6%)	27 (6.6)	
Asian	5 (0.9%)	6 (1.5)	
Other	7 (1.3%)	5 (1.2)	
Smoking history at time of analyzed CT			0.001
Current	123 (27.3%)	82 (24.3)	
Former	273 (60.7%)	237 (70.3)	
Never	54 (12.0%)	18 (5.3)	
FEV_1 (% predicted; 276 unknown)	82 (68, 96)	80 (64, 94)	0.300
DLCO (% predicted; 337 unknown)	69 (59, 83)	81 (70, 97)	<0.001
Histology of NSCLC tumor			< 0.001
Adenocarcinoma	360 (67.9)	225 (54.0)	
Squamous cell carcinoma	112 (21.2)	139 (33.3)	
Other	58 (10.9)	53 (12.7)	
Pathological stage of NSCLC tumor			0.015
I	285 (60.9%)	179 (50.3)	
II	98 (20.9%)	102 (28.7)	
III	46 (9.8%)	45 (12.6)	
IV	39 (8.3%)	30 (8.4)	
Body mass index	26.7 (23.1, 30.8)	27.3 (24.6, 30.6)	0.050
Body fat area in analyzed CT at L3 level			
Subcutaneous	22,169 (15,044, 31,007)	16,399 (12,468, 22,887)	< 0.001
Visceral	13,554 (8,258, 19,990)	24,292 (30,478, 55,315)	< 0.001
Total	37,452 (25,091, 50,683)	42,228 (30,478, 55,315)	< 0.001
Visceral fat index	0.38 (0.30, 0.45)	0.58 (0.50, 0.64)	< 0.001

[a]Number and percentage among known values, and median and inter-quartile range values are shown for categorical and continuous variables, respectivel. *CT*, computerized tomography; *DLCO,* diffusion capacity of lungs for carbon monoxide; *FEV_1*, forced expiratory volume at 1 second; *NSCLC*, non-small cell lung carcinoma.

[b]Fisher's exact and Welch t tests for categorical and continuous variables, respectively.
